# The increase of photosynthetic carbon assimilation as a mechanism of adaptation to low temperature in *Lotus japonicus*

**DOI:** 10.1038/s41598-018-37165-7

**Published:** 2019-01-29

**Authors:** Pablo Ignacio Calzadilla, Juan Manuel Vilas, Francisco José Escaray, Fernando Unrein, Pedro Carrasco, Oscar Adolfo Ruiz

**Affiliations:** 1 Instituto de Investigaciones Biotecnológicas-Instituto Tecnológico de Chascomús (IIB-INTECH), UNSAM-CONICET, Buenos Aires, Argentina; 2 Departamento de Bioquímica y Biología Molecular-Universitat de València, Valencia, Spain; 3 Instituto de Fisiología y Recursos Genéticos Vegetales (IFRGV) Ing “Victorio S Trippi”, Instituto Nacional de Tecnología Agropecuaria (INTA), Córdoba, Argentina; 4 Present Address: Institut de Biologie Intégrative de la Cellule (I2BC), CNRS, CEA, Université Paris-Sud, Université Paris-Saclay, Gif sur Yvette, France

## Abstract

Low temperature is one of the most important factors affecting plant growth, it causes an stress that directly alters the photosynthetic process and leads to photoinhibition when severe enough. In order to address the photosynthetic acclimation response of *Lotus japonicus* to cold stress, two ecotypes with contrasting tolerance (MG-1 and MG-20) were studied. Their chloroplast responses were addressed after 7 days under low temperature through different strategies. Proteomic analysis showed changes in photosynthetic and carbon metabolism proteins due to stress, but differentially between ecotypes. In the sensitive MG-1 ecotype acclimation seems to be related to energy dissipation in photosystems, while an increase in photosynthetic carbon assimilation as an electron sink, seems to be preponderant in the tolerant MG-20 ecotype. Chloroplast ROS generation was higher under low temperature conditions only in the MG-1 ecotype. These data are consistent with alterations in the thylakoid membranes in the sensitive ecotype. However, the accumulation of starch granules observed in the tolerant MG-20 ecotype indicates the maintenance of sugar metabolism under cold conditions. Altogether, our data suggest that different acclimation strategies and contrasting chloroplast redox imbalance could account for the differential cold stress response of both *L*. *japonicus* ecotypes.

## Introduction

Suboptimal temperature is one of the most important abiotic factors limiting plant growth. Frost have a significant impact on agricultural production, depending the extent of damages on factors such as the inherent tolerance of the plant, the severity and duration of the stress, whether it is repeated and whether it occurs in combination with other stressors. When exposed to low but non-freezing temperatures, many plants initiate a cold acclimation process that improves their ability to withstand freezing temperatures^1^.

Cold acclimation can be defined as the modifications of anatomy, physiology and metabolism that occur in response to sub-optimum temperatures that minimize irreversible freeze-damage and improve plant fitness. This process implies massive changes in gene expression^2,3^, enzymatic activities^4^, proteomic changes^5^ and subcellular reprogramming of metabolism^6^.

Although the mechanisms behind cold acclimation are not fully understood, it is known that photosynthetic responses to cold but non-freezing temperature can be important. Photosystems, electron transport chain, as well as the Calvin-Benson cycle and other carbon metabolisms reactions, are involved in the production of carbohydrates during photosynthesis. This complex process is finely regulated through different signals, allowing its homeostasis under different environmental conditions. However, its deregulation reduces CO_2_ fixation capacity and induces photoinhibition^7^. In general terms, the response to photoinhibition involves different photoprotective mechanisms which avoid a redox imbalance into the electron transport chain, chloroplast ROS generation and protein photodamage^8^.

Under adverse environmental conditions that restrict the rate of photosynthesis, photon capture can exceed the rate at which the energy can be used, resulting in production of reactive oxygen species (ROS) and cell damage^9,10^. It has been shown that low temperatures are directly related to ROS increase^11,12^, and a positive correlation has been observed between detoxification capacity of these molecules and cold stress tolerance^13,14^. In addition, it has been suggested that an efficient electron sink has the potential to counterbalance the excess of light energy absorption^15^. In consequence, an increase in carbon assimilation could be strongly relevant in the photosynthetic acclimation capacity and in the avoidance of chloroplast photo-oxidative stress in plants^16^.

*Lotus japonicus* is commonly used as a model legume for plant research, specifically concerning plant responses to stress^17,18^. Recently, we have suggested that a cold-induced redox imbalance is generated in two *L*. *japonicus* ecotypes (MG-1 and MG-20), with contrasting photosynthetic adaptability to low temperatures^19^. The differential response of MG-1 and MG-20 to low temperature conditions has been previously described^19^. After 7 days of chilling, the tolerant ecotype (MG-20) exhibited a better photosynthetic performance than the sensitive one (MG-1) and higher stomatal conductance, suggesting chloroplast involvement in this proccess^19^. However, the degree of participation of these organelles in the cold stress response in legumes is poorly studied.

Global proteomic analysis of rice leaves subjected progressively to low temperature^20^ and chloroplast proteome analysis against drought stress and recovery in tomato^21^ lead to the conclusion that changes in chloroplast proteins occur in the response and acclimation to abiotic stress. Thus, in the present study we aimed to evaluate the role of chloroplast in the *L*. *japonicus* low temperature response, by studying chloroplast stress-responsive proteins under cold stress using a proteomic approach. In addition, confocal microscopy and flow cytometry have been used to identify and quantify chloroplastic ROS generation. We hypothesize that different chloroplast strategies are involved in plant tolerance to cold stress.

## Results

### Proteomic analysis of *L*. *japonicus* chloroplasts under low temperature

**Figure 1:**
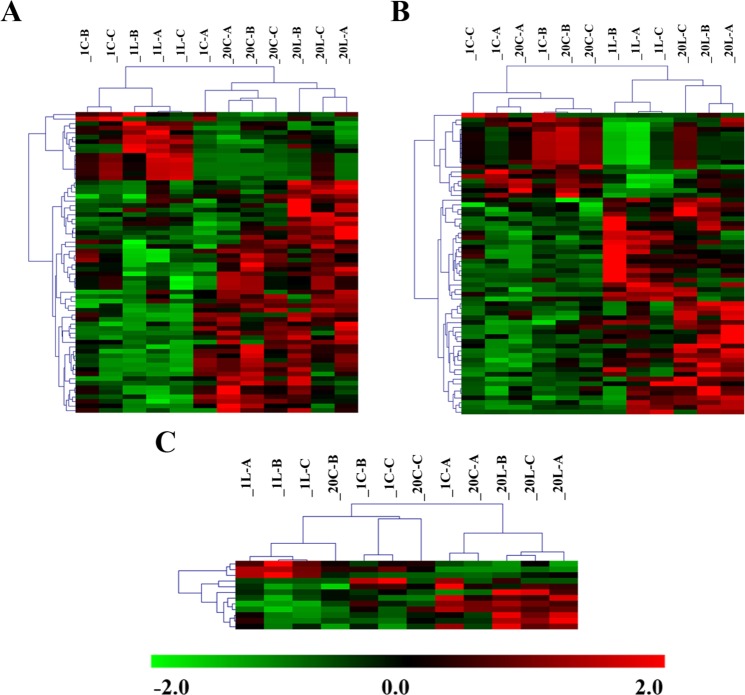
Hierarchical clusters of significant abundant proteins. The protein abundance is shown as a colour code, from green (lower abundance) to red (higher abundance). (**A**) Hierarchical cluster based on abundance differences between ecotypes (p < 0.05). (**B**) Hierarchical cluster based on abundance differences between control and low temperature treatment (p < 0.05). (**C**) Hierarchical cluster based on abundance differences of proteins with significative interaction between factors (p < 0.05). Three biological replicates are represented for each treatment, in each graph. 1C, MG-1 Control; 1L, MG-1 Low temperature; 20C, MG-20 Control; 20L, MG-20 Low temperature.

A discriminant analysis of the chloroplast proteome was carried out in the MG-1 and MG-20 *L*. *japonicus* ecotypes exposed to low temperature. Hierarchical analysis of the 724 identified proteins allowed us to group the differentially abundant protein in three clusters: 66 proteins differentially abundant only between ecotypes (Fig. 1A), 64 proteins differentially abundant due to low temperature (Fig. 1B) and 12 of them presenting interaction between ecotype and temperature factors (Fig. 1C).

The STRING software (version 10.0) was used to estimate functional association networks between the different identified proteins^22^. Networks were generated according to data from gene co-occurrence, co-expression, curated databases, experimentally determined and textmining, and the differentially abundant proteins between ecotypes and treatments were analysed. When comparing ecotypes, MG-1 chloroplasts showed a greater abundance of proteins related with the “light-reactions” of photosynthesis (in particular, photophosphorylation and antenna proteins) than chloroplast of the MG-20 ecotype (Supplementary Fig. 1A). In contrast, proteins related with nitrogen and carbon metabolism, highlighting saccharose and starch metabolism were more abundant in the MG-20 ecotype. As well, other proteins related with the oxidative stress response, protein folding and translation were also over-represented (Supplementary Fig. 1B).

**Figure 2:**
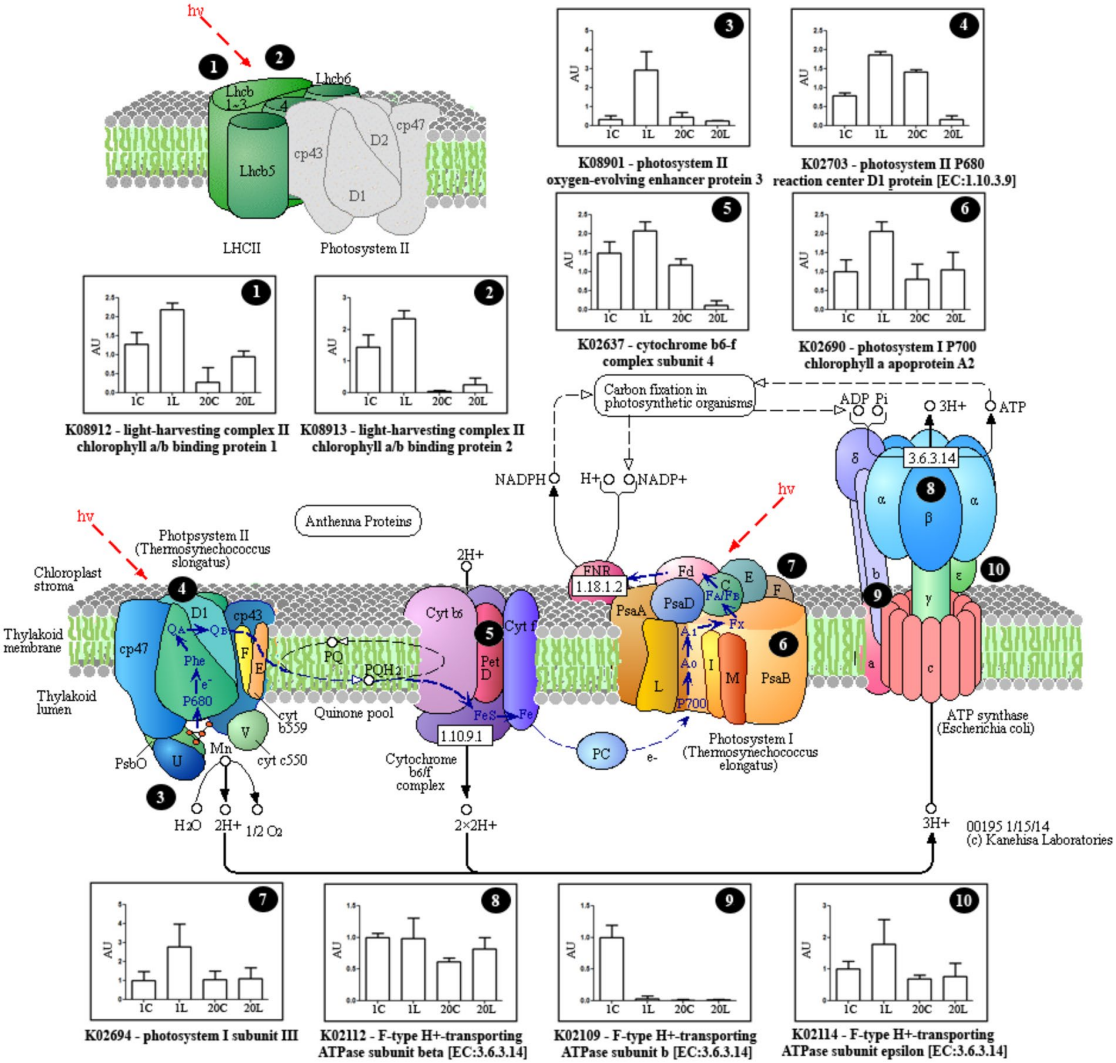
Differentially abundant proteins related with photosystems and the electron transport chain. Representative scheme showing the different identified proteins and their abundance. Values are the mean ± SD of three biological replicates, and are expressed as arbitrary units (AUs) relative to the MG-1 control treatment. 1C, MG-1 Control; 1L, MG-1 Low temperature; 20C, MG-20 Control; 20L, MG-20 Low temperature. Adapted scheme from the KEGG database (http://www.genome.jp/kegg/pathway/map/map00195.html)^63^.

**Figure 3:**
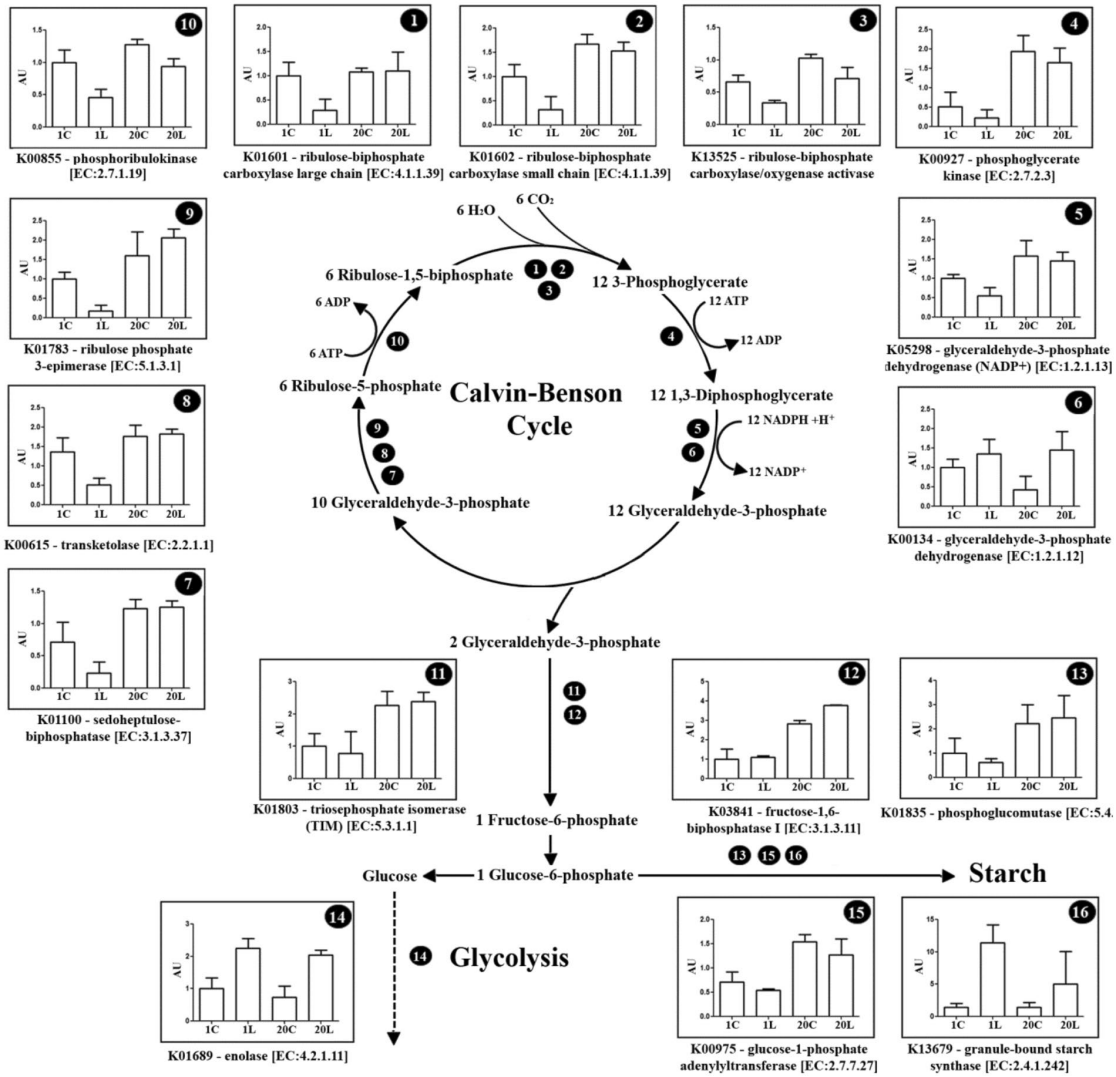
Differentially abundant proteins related with the Calvin-Benson cycle and the carbon metabolism. Representative scheme showing the different identified proteins and their abundance. Values are the mean ± SD of three biological replicates, and are expressed as arbitrary units (AUs) relative to the MG-1 control treatment. 1C, MG-1 Control; 1L, MG-1 Low temperature; 20C, MG-20 Control; 20L, MG-20 Low temperature.

**Figure 4:**
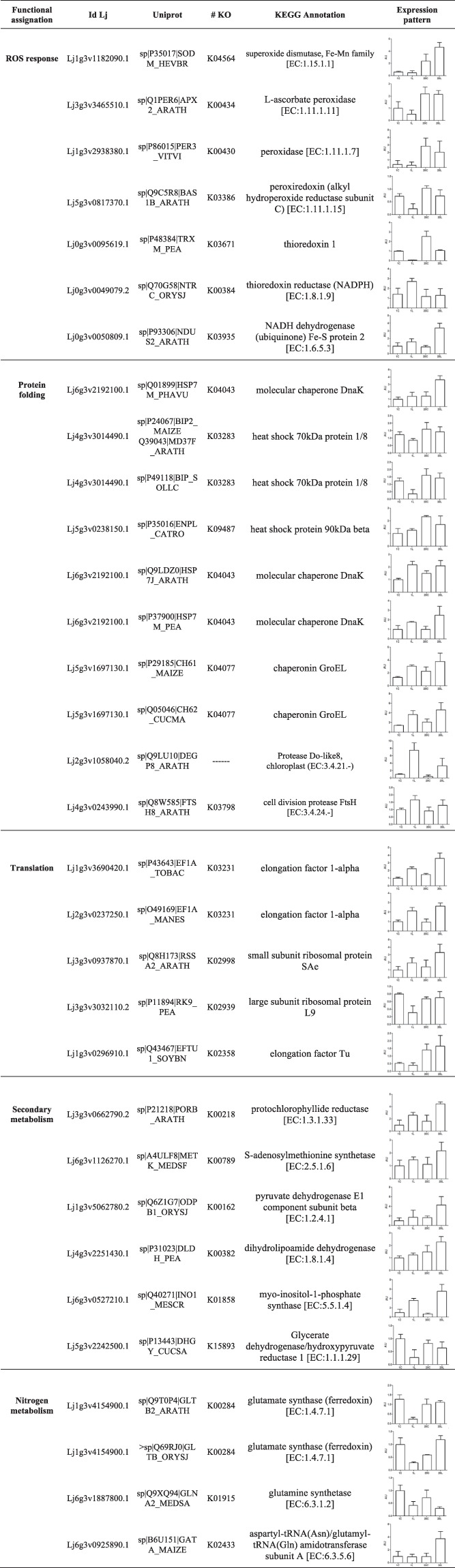
Functional assignation of the differentially abundant proteins in *L. japonicus* chloroplasts under low temperature. The classification and functional assignation of the significant abundant proteins (p < 0.05) was done using KEGG, LegumeIP and Uniprot databases. Values are the mean ± SD of three biological replicates, and are expressed as arbitrary units (AUs) relative to the MG-1 control treatment. 1C, MG-1 Control; 1L, MG-1 Low temperature; 20C, MG-20 Control; 20L, MG-20 Low temperature.

Proteins differentially abundant depending on temperature are clustered in Supplementary Fig. 2. Under control conditions, a low number of proteins were grouped into the glyoxylate pathway and carbon metabolism (Supplementary Fig. 2A). Nevertheless, a greater functional association network was observed as consequence of low temperature. Proteins related with secondary metabolism, photosynthetic processes, photo-phosphorylation, oxidative stress responses as well as translation, protein folding and degradation were over-represented in these networks (Supplementary Fig. 2B). All proteins used to estimate functional association networks are detailed in Figs 2–4. No functional associations were observed for proteins with significant interactions between ecotypes and the stress treatment (Fig. 1C, Supplementary Table 1).

### Differential changes in proteins belonging to photosystems and the electron transport chain

Analysis of the chloroplast proteome showed that the abundance of some proteins related to photosystems and the electron transport chain was different between the MG-1 and the MG-20 ecotypes, what could be related to their response to cold stress. Firstly, the photosystem II (PSII) light-harvesting complex II chlorophyll a/b binding proteins 1 and 2 (Lhcb1 and Lhcb2), increased their abundance as consequence of cold stress in both ecotypes (Fig. 2). However, the levels of these proteins were higher, even under control conditions, in the sensitive MG-1 ecotype (Fig. 2). A contrasting effect was observed in the stress response of the photosystem II (PSII) components, oxygen-evolving protein 3 (OEE3) and D1 protein, and the cytochrome *b6f* complex subunit 4. While their levels were increased in MG-1 plants as a consequence of cold exposure, they were reduced in the MG-20 tolerant ecotype (Fig. 2). The identified photosystem I (PSI) proteins, P700 chlorophyll *a* apoprotein A2 and subunit III, raised their abundance as consequence of cold stress in a similar way to the PSII Lhcb proteins, although the abundance of these proteins was almost not affected by stress in the tolerant ecotype (Fig. 2).

Meanwhile, three subunits of the chloroplast ATP synthase [EC:3.6.3.14] (β, ε and *b*) were also identified (Fig. 2). Protein levels of the subunit β were increased under low temperature in MG-20, while no differences were observed between treatments in MG-1 plants. On the contrary, higher levels of subunit ε were registered under stress in MG-1, but no differences were observed in MG-20 between treatments. Regarding the *b* subunit, their protein abundance was only reduced in the sensitive MG-1 ecotype under low temperature. No differences between treatments were observed in MG-20, although in both cases its protein levels were lower than those in MG-1.

### Differentially abundant proteins related with Calvin-Benson cycle, gluconeogenesis and starch biosynthesis

In general, proteins involved in Calvin-Benson cycle were more abundant in the tolerant ecotype MG-20 (Fig. 3). However, as a consequence of low temperature, the level of some of those enzymes was reduced, especially in the MG-1 sensitive ecotype (Fig. 3). Differences to cold response were observed between ecotypes for the following proteins: the large and small subunit of ribulose-1,5-bisphosphate decarboxylase (Rubisco) [EC:4.1.1.39], Rubisco activase, phosphoglycerate kinase [EC:2.7.2.3] and a NADP^+^ dependent glyceraldehyde-3-phosphate dehydrogenase [EC:1.2.1.13] (Fig. 3). In addition, a reduction in the levels of sedoheptulose 1,7-bisphosphatase [EC:3.1.3.37], transketolase [EC:2.2.1.1] and ribulose-phosphate 3-epimerase [EC:5.1.3.1] were observed under stress in MG-1 (Fig. 3), while no differences were observed between treatments in the MG-20 line. Similar results were obtained for the phosphoribulokinase enzyme [EC:2.7.1.19], although in this case its levels were also reduced in the tolerant ecotype under low temperature (Fig. 3). A NAD^+^ dependent glyceraldehyde-3-phosphate dehydrogenase isoform **[**EC:1.2.1.12] was also identified, but this protein increased its abundance under low temperature in both ecotypes.

Regarding gluconeogenesis proteins, chloroplast isoforms of the triosephosphate isomerase [EC:5.3.1.1] and the fructose-1,6-bisphosphatase were also identified [EC:3.1.3.11] (Fig. 3). The abundance of these proteins was higher in the MG-20 tolerant ecotype, being the fructose-1,6-bisphosphatase levels also increased under stress only in this line. Simultaneously, the levels of enolase [EC:4.2.1.11] increased under low temperature in both ecotypes (Fig. 3).

The abundance of at least three proteins involved in the starch biosynthetic pathway, phosphoglucomutase (PGM, [EC:5.4.2.2]), glucose-1-phosphate adenylyltransferase (AGPase, [EC:2.7.7.27]) and granule-bound starch synthase (GBSS, [EC:2.4.1.242]) was affected by cold (Fig. 3). The PGM and AGPase proteins were more abundant in MG-20 ecotype under both conditions. However, GBSS levels were higher in the sensitive ecotype, only under stress (Fig. 3).

### Identification of other proteins participating in the low temperature response

Beyond proteins related to photosynthesis and sugar metabolism, proteomic analysis allowed us to identify a number of proteins whose abundance also differed among ecotypes as a consequence of cold stress (Fig. 4).

The tolerant MG-20 ecotype showed higher levels of proteins involved in antioxidant responses and ROS scavenging, such as superoxide dismutase [EC:1.15.1.1], ascorbate peroxidase [EC:1.11.1.11], peroxidase enzymes [EC:1. 11.1.17], protein thioredoxin 1 and peroxiredoxin [EC:1.11.1.15], and NADH dehydrogenase [EC:1.6.5.3]. In general, the abundance of these proteins was higher in the tolerant ecotype in control and stress conditions, when compared to MG-1 (Fig. 4). On the contrary, thioredoxin reductase [EC:1.8.1.9] was the only protein involved in ROS response which levels increased in the MG-1 ecotype under stress, while no differences were observed in MG-20 between treatments (Fig. 4).

Four heat shock proteins (Hsps) and five chaperones were also identified in the proteomic analysis (Fig. 4). Except for the Hsp 70 kDa and 90 kDa, all other proteins showed an increase in their levels under stress, compared to their abundance in the control condition, in both ecotypes. Similar results were observed for translation related proteins. Elongation factor 1-α (*Lj1g3v3690420*.*1*) and the predicted 40S ribosomal protein (*Lj3g3v0937870*.*1*) were more abundant in the tolerant ecotype in comparison with MG-1 (Fig. 4). Other proteins as the elongation factor EF-Tu (*Lj3g3v3032110*.*2*) and the large ribosomal subunit L9 (*Lj3g3v3032110*.*2*), showed no increment in their abundance under low temperature conditions. However, a higher protein level was found in the MG-20 ecotype under this condition (Fig. 4).

Furthermore, the levels of protochlorophyllide reductase [EC:1.3.1.33] and S-adenosylmethionine synthetase [EC:2.5.1.6] were increased under low temperature in both ecotypes, but to a higher extent in MG-20 than in MG-1 (Fig. 4). Similar results were observed for the pyruvate dehydrogenase E1 (β subunit) [EC:1.2.4.1], the dihydrolipoamide dehydrogenase [EC:1.8.1.4] and the myo-inositol-1-phosphate synthase [EC:5.5.1.4] (Fig. 4). On the contrary, a decreased in the abundance of some proteins related with the glyoxylate metabolism was observed under stress condition. In particular, this effect was evidenced for the glutamine synthetase [EC:6.3.1.2] and the glycerate dehydrogenase/hydroxypyruvate reductase [EC:1.1.1.29] (Fig. 4). In the latter case, the reduction was more pronounced in the MG-1 ecotype.

Regarding the nitrogen metabolism, in addition to the glutamine synthetase, the glutamate synthase [EC:1.4.1.7] protein levels were also reduced under low temperature, but only in the sensitive ecotype (Fig. 4). Moreover, no differences in glutamyl-tRNA (Gln) amidotransferase [EC:6.3.5.6] protein levels were observed between treatments in MG-1, while an increased was evidenced in the tolerant ecotype under stress (Fig. 4).

### Ultrastructural modifications of *L*. *japonicus* chloroplasts under low temperature

**Figure 5:**
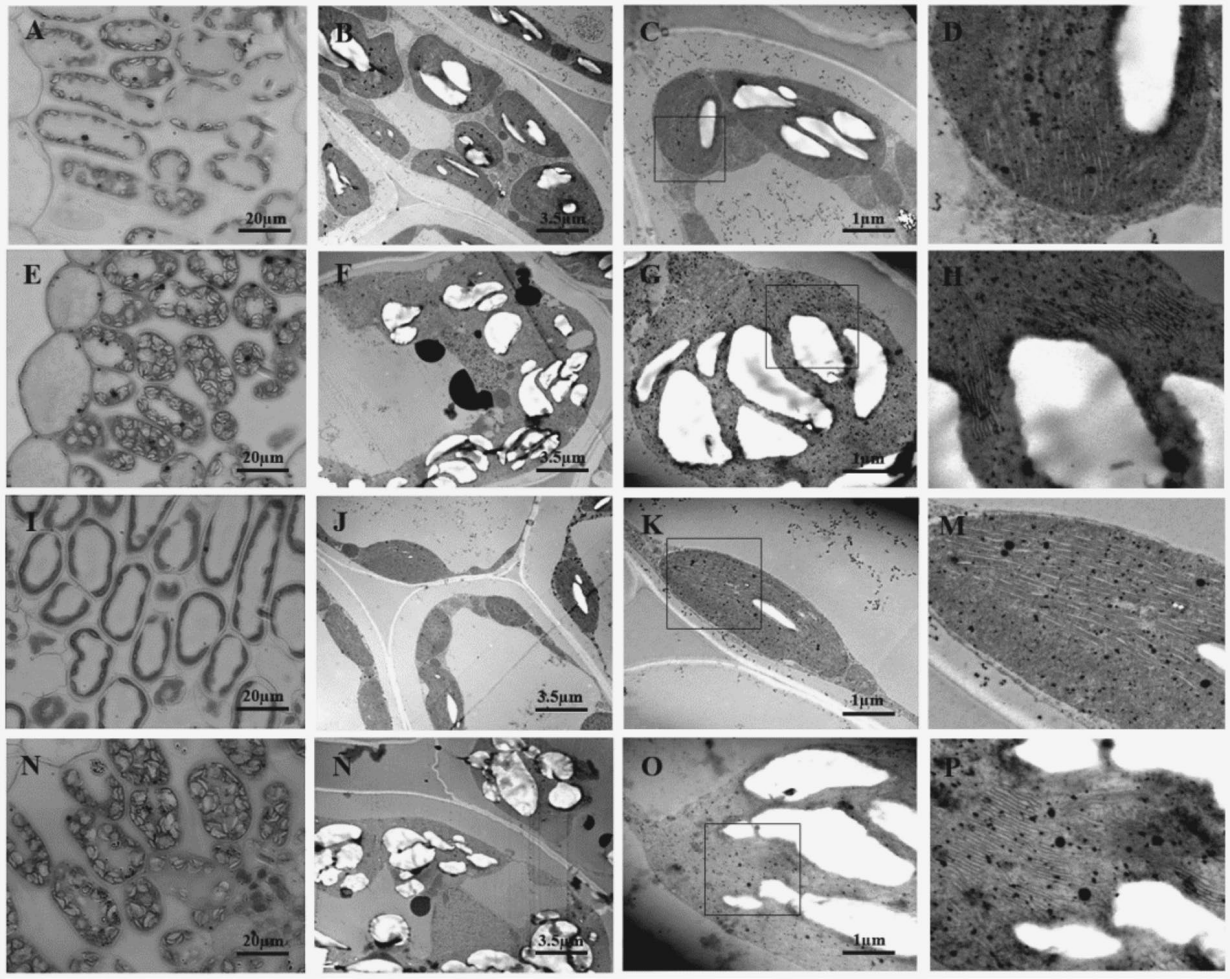
Micrographs of *L. japonicus* leaves under 7 d of low temperature and control treatments. Representative images obtained by optical and transmission electron microscopes. (**A**–**D)** MG-1 Control. (**E**–**H**) MG-1 Low temperature. (**I**–**K**,**M**) MG-20 Control. (**N**,**Ñ**,**O**,**P**) MG-20 Low temperature. The squares in images (**C**,**G**,**K**,**O**) delimit the amplified area in (**D**,**H**,**M**,**P**).

Chloroplasts of MG-1 and MG-20 ecotypes under control and low temperature conditions were observed by transmission electron microscopy. Under cold stress, the chloroplast area and the amount of starch granules increased in both ecotypes, being this increase higher in the MG-20 tolerant line (Fig. 5 and Table 1). Moreover, a higher number of electron-dense particles were registered under low temperature in the chloroplasts of both ecotypes (Fig. 5D,H,M,P). However, their density was higher in the MG-1 ecotype under stress (Fig. 5C,D,G,H,K–M,O,P).

**Table 1:** Ultrastructural parameters of *L. japonicus* chloroplasts.

*Ultrastructural Parameters*	MG-1		MG-20	
	C	L	C	L
Chloroplasts area (µm^2^)	9.9 ± 3.5	27.2 ± 6.6^*^	10.6 ± 3.2	38.3 ± 3.6^*^
N° Starch granules	1.8 ± 1.0	5.2 ± 1.3^*^	0.8 ± 1.0	6.4 ± 2.1^*^
Starch granules area (µm^2^)	1.0 ± 0.6	2.0 ± 1.1	0.4 ± 0.3	3.1 ± 1.4^*^

Values are the mean ± SD of three biological replicates, for each treatment. The asterisk indicates differences between control and low temperature treatments (Student’s test, p < 0.05), for each ecotype respectively. C, Control; L, Low temperature.

In addition, it was no possible to distinguish chloroplast grana under control conditions, although alterations in the thylakoid membranes were observed in response to stress in both ecotypes. These disruptions were more evident in the MG-1 ecotype (Fig. 5D,H,M,P). Furthermore, a reduction in the vacuole area of the cells was also observed as a consequence of stress (Fig. 5A,E,I,N).

### Determination of chloroplast ROS production under low temperature

**Figure 6:**
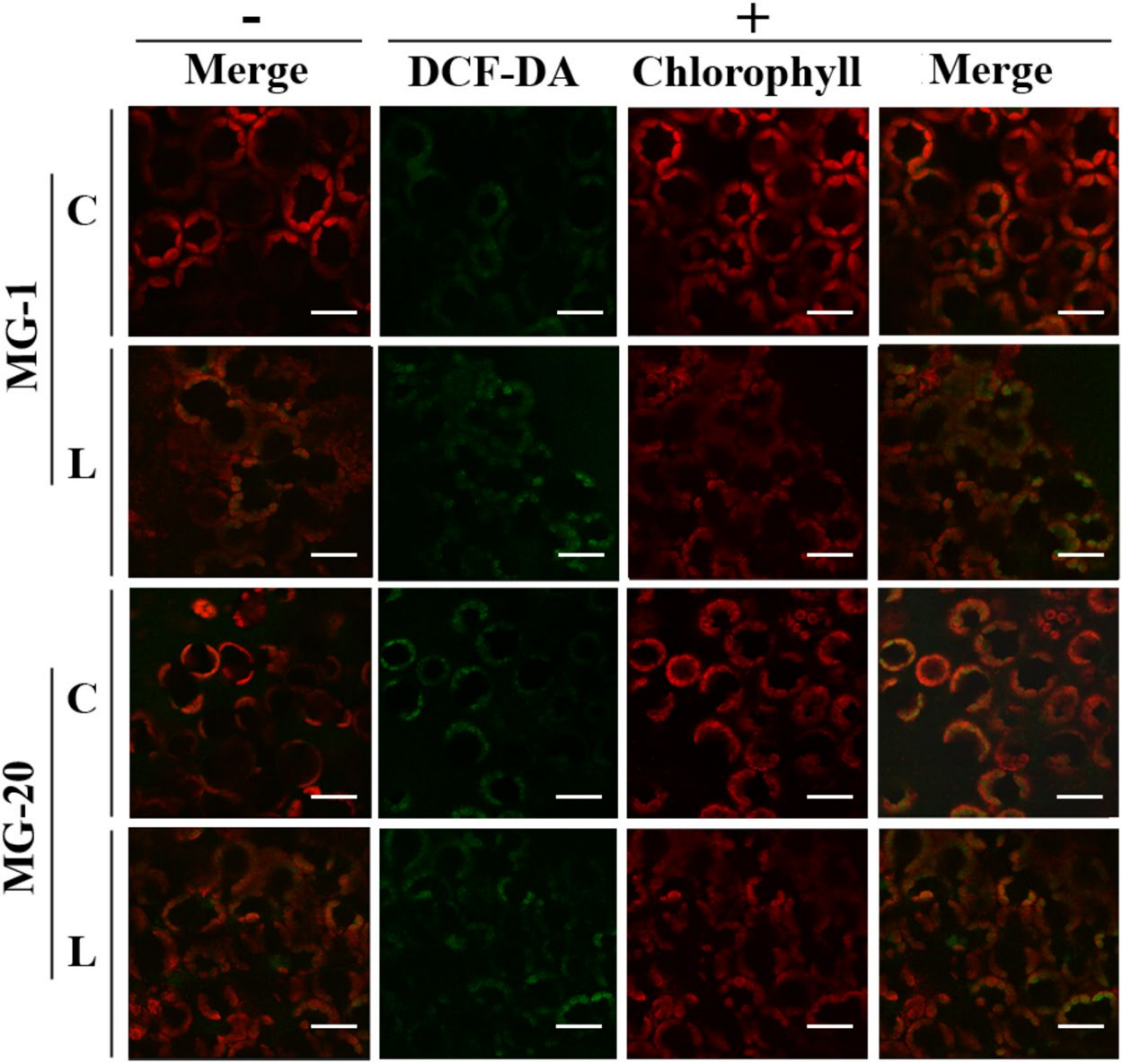
ROS subcellular localization in *L. japonicus* leaves. Representative images from confocal laser microscopy showing subcellular localization of the DCF-DA fluorescence (green) and chlorophyll autofluorescence (red) in MG-1 and MG-20 ecotypes. C, Control; L, Low temperature; −, DCF-DA incubation without light; +, DCF-DA incubation with light. Scale bars = 30 µm.

Co-localization of the DCF-DA fluorescence (ROS marker, green) and chlorophyll auto-fluorescence (chloroplasts marker, red) was observed in both ecotypes by confocal microscopy (Fig. 6). No differences due to low temperature were observed in the green fluorescence intensity in MG-20. However, a higher green fluorescence was registered under stress in MG-1 (Fig. 6). When comparing both ecotypes, the tolerant MG-20 ecotype exhibited higher levels of ROS under control conditions than MG-1 plants (Fig. 6). Meanwhile, chlorophyll auto-fluorescence decreased in both ecotypes after cold exposure (Fig. 6). In the absence of DCF-DA, no green fluorescence was registered in *L*. *japonicus* leaves observed under the confocal microscope (Supplementary Fig. 3).

In order to quantify the differences observed by confocal microscopy, chloroplast ROS production was determined by flow cytometry in isolated cells. Two cytometric populations (*a* and *b*), obtained from *L*. *japonicus* leaves, were identified based on differences in their chlorophyll autofluorescence (FL3) and their scattered light (SSC) (Supplementary Fig. 4). Epifluorescence microscopy allowed the identification of chloroplasts and nucleus in *a*, demonstrating that was composed mainly by whole cells (Supplementary Fig. 4C,D). Due to its cell integrity, flow cytometry was performed on the *a* population.

**Figure 7:**
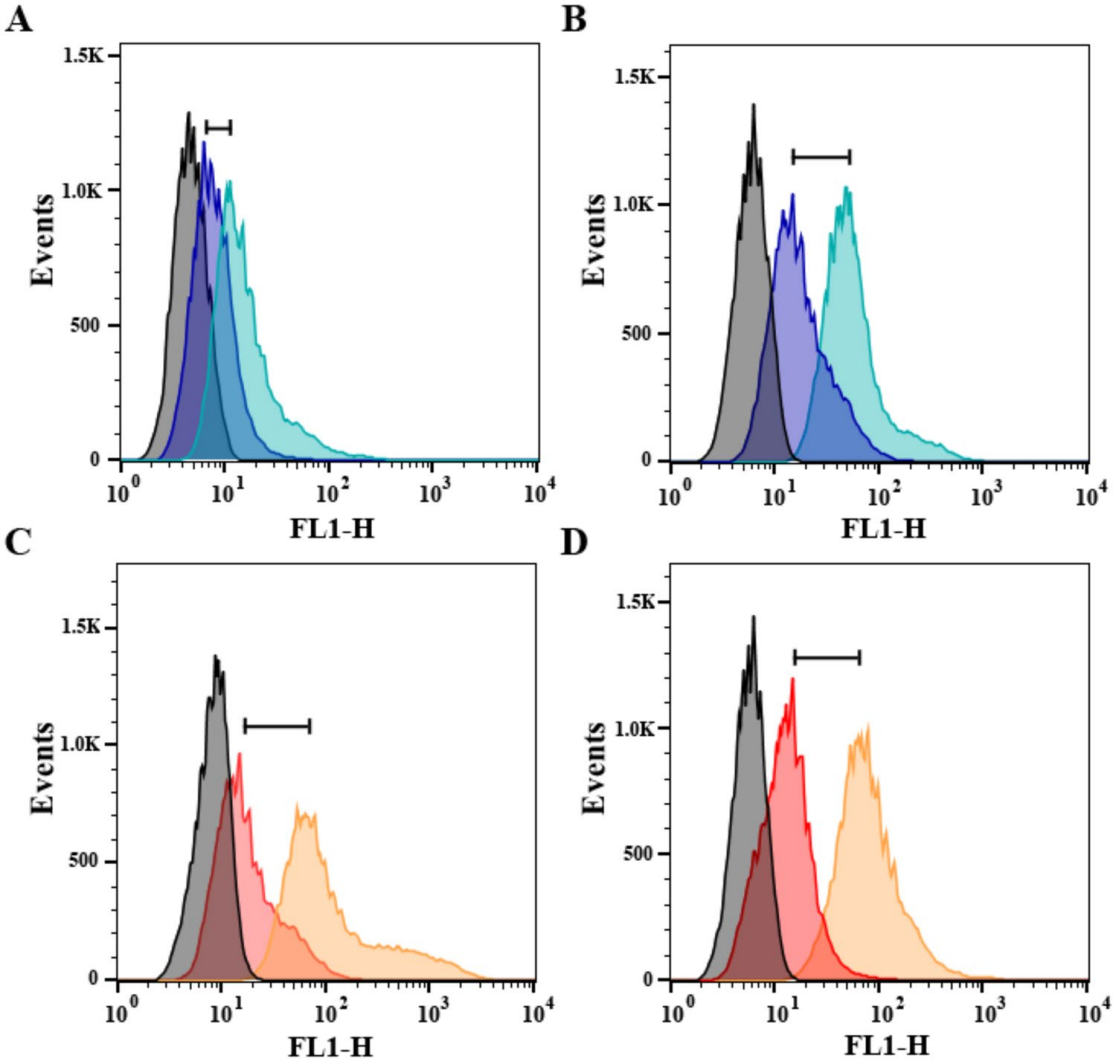
Representative histograms showing FL1 fluorescence intensity of cells population of *L. japonicus* leaves under low temperature. (**A**,**B**) Histograms of MG-1 cells under control and low temperature treatment, respectively. Blue histogram: incubation with DCF-DA without light. Light blue histogram: incubation with DCF-DA and light. (**C**,**D**) Histograms of MG-20 cells under control and low temperature treatment, respectively. Red histogram: incubation with DCF-DA without light. Orange histogram: incubation with DCF-DA and light. Grey histograms: incubation without DCF-DA or light. A total of 40.000 events were analyzed in each case.

**Figure 8:**
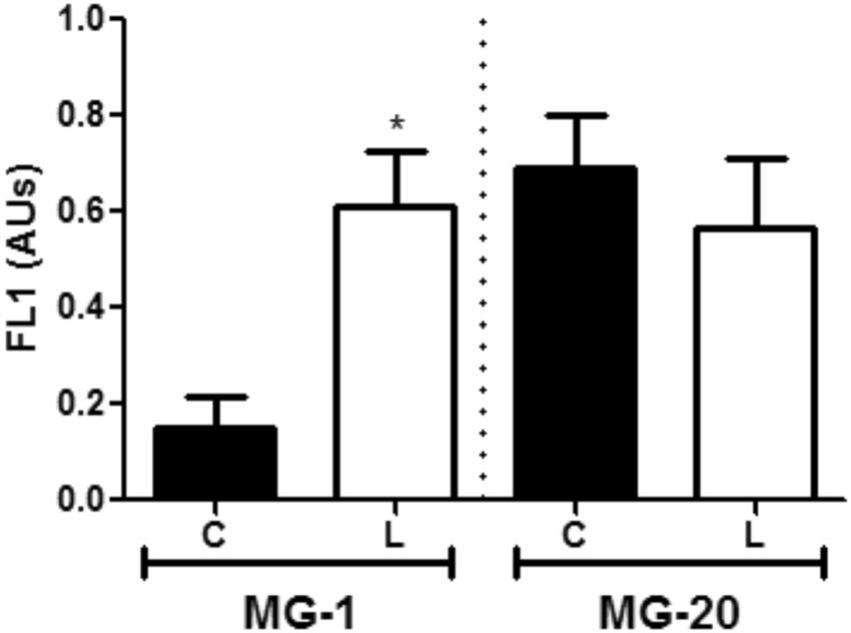
ROS production quantification by flow cytometry. The DFC-DA fluorescence intensity (FL1) was calculated in each case using the formula in Materials and Methods. Values are the mean ± SD of 5 biological replicates and are expressed as arbitrary units (AUs), for each ecotype and treatment. The asterisk indicates differences between control and low temperature treatments (Student’s test, p < 0.05), for each ecotype respectively. C, Control; L, Low temperature.

All cells incubated in the absence of DCF-DA showed the same FL1 relative fluorescence (DCF-DA fluorescence) (Fig. 7, and Supplementary Fig. 4). When cells were incubated with DCF-DA but without light exposure, a similar increase in green fluorescence was observed in all populations (Fig. 7, Supplementary Fig. 4). However, treatments with DCF-DA and light exposure showed a higher FL1 fluorescence intensity in cells exposed to cold only in the MG-1 sensitive ecotype (Fig. 7 and Supplementary Fig. 4). In this sense, relative differences between FL1 mode values suggest an increment of almost two-fold in MG-1 ROS levels under stress, compared to its control conditions (Fig. 8). Meanwhile, as observed by confocal microscopy, no significant increment in ROS levels was detected after low temperature treatment in the MG-20 tolerant ecotype. Nevertheless, ROS levels under control conditions were higher in the tolerant ecotype (Figs 7 and 8).

## Discussion

Plants response to different environmental stimuli involves complex transcriptional, protein and metabolic changes^23^. In particular, chloroplasts participate in stress sensing and signalling through changes in their redox state and their direct implication in photosynthesis and carbon metabolism^24^. Increasing evidence supports a chloroplastic role in the response and acclimation to abiotic stress in plants^25^. Moreover, transcriptomic changes produced by cold stress in *L*. *japonicus* also suggest chloroplast involvement in the response to low temperature in this species^3^. In this work, we provide novel insights into the cold stress response in *L*. *japonicus* by analysing changes in chloroplastic proteins abundance, which are associated with ROS production and photoinhibition.

### Proteomic changes in *L*. *japonicus* chloroplast in response to low temperature

We have compared the behaviour of two ecotypes with differential tolerance to low temperature: MG-1 and MG-20. We identified 66 proteins differentially abundant between them (p < 0.05), regardless of the temperature treatment to which they were subjected (Fig. 1A). These differences could be explained due to the genetic variability of the ecotypes^26^. In addition, approximately 10% of the total identified proteins showed significant changes in their abundance as consequence of low temperature (p < 0.05) (Fig. 1B). Functional assignment and classification of all the differentially abundant proteins allowed the identification of metabolic pathways over-represented in each ecotype (Supplementary Fig. 1), and altered by cold stress (Supplementary Fig. 2). Our results suggest that differences in the chloroplast proteome between MG-1 and MG-20 plants could be related with their differential tolerance to low temperature. Characterization of chloroplast proteome in response to drought stress in tomato^21^ and in salt-stressed mangrove^27^ lead to the identification of a number of proteins involved in abiotic stress responses that have been also identified in *L*. *japonicus* (Supplementary Figs 1 and 2). Altogether, these results suggest that chloroplast is involved in plant responses to cold stress.

### Energy dissipation in the thylakoid membranes

An increase in Lhcb1 and Lhcb2 (LHCII proteins) levels was observed mainly in chloroplasts of the MG-1 plants in response to low temperature (Fig. 2). Beyond solar light absorption, the LHCII complex has also a key role in the regulation, distribution and dissipation of excessive excitation energy under photoinhibitory conditions, avoiding photodamage^28^. In particular, Lchb1 and Lhcb2 proteins play an important regulatory role in the photoprotective mechanism called Non-Photochemical Quenching (NPQ)^29^. Other components of the LHCII complex (Lhcb4 and Lhcb6) have also been described as differentially expressed in mangrove chloroplast as a consequence of salt stress^27^.

In addition, an increase in chloroplast ROS production, ELIP1 protein and the thylakoidal luminal 19 kDa protein levels were observed in MG-1 plants under the low temperature treatment (Figs 2 and 8). ELIP1, a member of the multigenic family of the light harvesting complex, is accumulated under high light and has been related with photoprotection under photo-oxidative conditions^30^. Meanwhile, although the thylakoid luminal protein 19 kDa has an unknown function, these proteins have also been linked to oxidative stress response in chloroplasts^31^. Together, these data supports that a cold-induced redox imbalance is generated in *L*. *japonicus* chloroplasts, and mainly in the sensitive MG-1 ecotype.

Furthermore, two PSII components were identified in our proteomic study: D1 reaction centre protein and OEE3 (PsbQ, OEC subunit) (Fig. 2). In particular, the D1 protein has been widely characterized as the main PSII photodamage target during photoinhibition^32^. OEE1 and OEC proteins were also induced in chloroplasts of mangrove under salt stress^27^ and maize^33^ and tomato^21^ under drought stress. Stress conditions produce an excess of excitation energy that damages D1 protein, initiating the PSII damage and repair cycle^8^. In this process, different chloroplast proteases are involved. Two of them, FtsH and Deg8, increased their levels under low temperature in *L*. *japonicus* (Fig. 4). Both proteins participate cooperatively in damaged D1 degradation^8^, suggesting activation of the PSII damage and repair cycle. These results are consistent with the photoinhibition previously reported under cold stress in both ecotypes^19^.

The OEE3 subunit has been related with PSII assembly^34^ and with the PsbO protein, which maintains the manganese cluster stability required for water oxidation^35^. While OEE3 protein levels were increased under low temperature in the sensitive ecotype MG-1, a reduction was observed in MG-20 plants under the same conditions (Fig. 2). Although these proteins seems to be relevant for PSII function and D1 protein repair cycle, it has been demonstrated that reduced levels of PsbO protein increase abiotic stress tolerance and reduce photoinhibition in transgenic potato plants^36^. Notwithstanding the photochemical mechanisms related with this tolerance increase are unknown, these results are consistent with the better acclimation of the MG-20 ecotype under cold stress.

Meanwhile, an increment in one subunit of the cytochrome complex *b6f* and two components of PSI was only observed in the sensitive ecotype under stress (Fig. 2). Both cytochrome *b6f* and PSI are strongly related to cyclic electron transport (CET)^37^, which participates in cold acclimation and is induced by an excess of photosynthetic electrons not utilized in carbon assimilation^38^. This data suggest that CET could be part of the cold-stress responses in *Lotus*.

### Carbon assimilation and starch biosynthesis under cold stress

Higher protein levels of Calvin-Benson cycle and sugar metabolism enzymes were observed in the tolerant ecotype MG-20 compared to MG-1, in response to cold stress (Fig. 3). Accumulation of proteins from these metabolic pathways (in particular Rubisco) has been related to cold stress tolerance in different plant species^39,40^ and directly with chloroplast responses to abiotic stress^21,33^. This effect could be explained by a higher electron flux through Calvin-Benson cycle, which could reduce excitation pressure on the electron transport chain components, avoiding photoinhibition^16^.

We have observed higher levels of sedoheptulose-1,7-bisphosphatase (SBPase) and fructose-1,6-bisphosphatase in the tolerant MG-20 ecotype under cold stress. It has been recently demonstrated the relevance of SBPase and fructose-1,6-bisphosphatase in the abiotic stress response in plants^33,41,42^. In particular, over-expression of SBPase has been linked to a reduction of the chilling-induce oxidative stress in transgenic tomato lines^42^. Similar results have been also observed in tobacco plants over-expressing fructose-1,6-bisphosphatase and exposed to saline stress^41^. Meanwhile, a higher accumulation and activity of the fructose-biphosphate aldolase was linked with cold acclimation in *Lolium multiflorum*/*Festuca arundinacea* introgression forms^40^. Although this enzyme was not identified in our proteomic study, it has been also pointed out the relevance of adjusting photosynthetic carbon assimilation to deal with low temperature stress in the mentioned forage grasses^40^. Altogether, these data lead to the conclusion that similar cold stress responses could be present in a wide range of species.

Regarding starch metabolism, three enzymes were identified: PGM, AGPase, and GBSS (Fig. 3). The reaction catalyzed by AGPase has been considered as a key step in starch biosynthesis^43^, although the PGM chloroplast isoform could also be relevant in this process^44^. Both enzymes showed higher protein levels in the tolerant ecotype (Fig. 3). In addition, an increase in GBSS protein levels (involved in amylose biosynthesis) was observed under cold stress in both ecotypes, suggesting starch composition changes in *L*. *japonicus* chloroplasts in response to stress. In fact, an increase in starch granules content was observed in both *L*. *japonicus* chloroplasts under the low temperature treatment, being this increment higher in the MG-20 ecotype (Fig. 5 and Table 1).

### Differential proteins involved in protein biosynthesis and folding

Complementary to photosynthesis and sugar metabolism, we found a number of proteins responding to cold stress in both ecotypes. Firstly, the DnaK and GroEL chaperones increased their levels in response to low temperature, and to a greater extent in the MG-20 ecotype compared to MG-1 (Fig. 4). These molecules have been related with abiotic stress tolerance in plants^21,45^. In particular, chloroplast chaperonins have been implicated in Rubisco biosynthesis^46^, what could suggest their involvement in the photosynthetic acclimation response to stress.

Among the proteins identified, we also found the 1-alpha and Tu elongation factors, which are components of the translation machinery in plants. Both factors have been related with abiotic stress responses, being the last particularly associated with PSII repair under cold stress^47,48^. Protein levels of both elongation factors were higher in MG-20 under low temperature (Fig. 4). This data could also support the better photosynthetic acclimation response previously reported in the tolerant ecotype^19^.

### ROS as a part of acclimation response to low temperature

Chloroplast ROS generation inhibits protein synthesis^49^ and could induce or increase the photoinhibitory effect under stress conditions^50^. Cold stress produces ROS generation and chloroplast redox alterations. The increase in ROS production is associated with a lower plant tolerance to restrictive conditions^13,14^. Thus, an increase in transcript and protein levels, as well as in the activity of different antioxidant enzymes, have been widely described as part of the cold stress response in plants^51^, and directly related with chloroplast ROS detoxification in different species^13,14^. As a consequence, higher protein abundance of different antioxidant enzymes in MG-20 chloroplasts, compared to MG-1, could explain its better acclimation to low temperature stress (Fig. 4). A higher cellular antioxidant capacity has been also related to cold stress tolerance in *L*. *multiflorum*/*F*. *arundinacea* species^40^.

Consistently, an increase in chloroplast ROS levels was observed under low temperature conditions in the MG-1 ecotype, while no changes were detected in MG-20 between treatments (Figs 6 and 8). In this sense, we observed alterations in the thylakoid membranes under cold stress (Fig. 5H,P) that could be related with chloroplast redox imbalance^52^. Disruption of thylakoid membranes was more evident in MG-1, which is consistent with the higher chloroplast ROS accumulation in this ecotype (Figs 6 and 8).

Moreover, the reduction in thioredoxin (Trx), peroxiredoxin and thioredoxin reductase (TrxR) protein levels, observed in both ecotypes under low temperature (Fig. 4), suggests that the Trx-TrxR system could be implicated in the *L*. *japonicus* chloroplast response to cold stress. The Trx-TrxR system participates in ROS detoxification, protein redox regulation, as well as in different signalling mechanisms^53^. In agreement with our results, down-regulation of thioredoxin gene expression by cold stress has been previously reported in plants^54^. Nevertheless, the fact that Trx-TrxR protein levels were higher in the tolerant MG-20 ecotype, could suggest a higher ROS detoxification capacity in the tolerant ecotype than in the sensitive one.

It is worth mentioning that higher ROS levels were observed in the tolerant ecotype, under control conditions (Figs 6 and 8). Although their cellular toxicity, these molecules have also been widely related with signalling mechanisms in plants^54^. As a consequence, the observed basal differences in ROS levels could suggest a differential regulation of the redox state and signalling, in response to stress, between ecotypes^55^. In addition, a regulatory role has been described for the Calvin-Benson cycle and carbon metabolism^56,57^. These results could imply a tighter regulation of the cold stress response in MG-20 than in MG-1, which would be consistent with its higher tolerance to this constrain condition^19^.

### Concluding remarks

Differences observed in the photosynthetic acclimation process of the *L*. *japonicus* MG-1 and MG-20 ecotypes could be explained, at least partially, by a differential chloroplast response. Higher abundance of proteins related with the antioxidant system, heat shock proteins and translation, suggest a better antioxidant response and lower cold-induced protein damage in the MG-20 ecotype. On the other hand, protein changes in the photosynthetic pathways as well as in carbon metabolism, suggest that two different acclimation strategies could be taken place in the chloroplast of the *L*. *japonicus* ecotypes studied. While in the MG-1 ecotype these mechanisms could involve mainly energy dissipation processes (such as NPQ and CET), the tolerant ecotype MG-20 would regulate carbon assimilation to cope with photoinhibition. The higher starch accumulation observed in the MG-20 chloroplasts, compared to MG-1, is consistent with these different acclimation strategies. In consequence, only the tolerant ecotype could efficiently balance the absorption and consumption of energy. This would explain the higher ROS levels observed in the MG-1 ecotype and its higher photoinhibition under these conditions. In conclusion, our results emphasize the relevance of the Calvin-Benson cycle and carbon metabolism in the photosynthetic acclimation process of *L*. *japonicus* under low temperature.

## Materials and Methods

### Plant materials, growth conditions and treatments

Seeds of *L*. *japonicus* MG-20 and MG-1 ecotypes were incubated for 7 d in a growth chamber with a 16/8 h day/night cycle at 24/21 ± 2 °C and 55/65 ± 5% relative humidity. Light (at an intensity of 250 µmol m^−2^ s^−1^) was provided by Grolux F 40 W fluorescent lamps. Seedlings were transferred to 110 mL volume pots containing sterilized sand-perlite (2:1), irrigated with 0.5X Hoagland’s nutrient solution^58^, and cultivated under the same temperature, relative humidity, and light conditions already described. In order to stress both ecotypes at the same developmental stage, plants with 4–6 fully developed leaves were used for all experiments.

The experimental design was completely randomized, with three biological repetitions per treatment. For both, the control and low temperature treatments, the 3-week old seedlings were placed in a Percival E-30B (Percival Scientific, Perry, IA, USA) growth chamber under a 16/8 photoperiod (day/night). Low temperature treatment was performed at 9/5 °C for 7 d, while control was performed at 24/21 ± 2 °C. Illumination was provided by Philips T8 Standard lamps (F17T8/TL841/ALTO 30PK), with an intensity of 250 µmol m^−2^ s^−1^. All the experimental determinations were done at 7 d of control and low temperature treatments, because it was the time when the higher photosynthetically acclimation differences were observed between ecotypes^19^.

### Chloroplast protein isolation

Chloroplasts isolation was performed as described previously^59^, with modifications. Detached leaves (1 g, approx. 5 plants) were homogenized in a mortar with 10 mL of homogenization buffer (50 mM HEPES pH 7.6, 330 mM sorbitol, 2 mM EDTA, 1 mM MgCl_2_, 5 mM ascorbic acid, 0.05% m/v BSA, 0.5 PMSF). The homogenate was filtered with gaze and centrifuged at 1,500 g for 7 min at 4 °C. The supernatant was discarded and the precipitate was re-suspended in 1 mL of homogenization buffer. Then, the suspended volume was transferred to a 15 mL Falcon tube with 10 mL of 30% Percoll in washing buffer solution (50 mM HEPES pH 8.0, 330 mM sorbitol, 2 mM EDTA, 1 mM MgCl_2_, 5 mM ascorbic acid, 0.05% m/v BSA, 0.5 mM PMSF). Samples were centrifuged at 5,000 g for 12 min at 4 °C, and the chloroplast enriched pellet collected.

The pellet was re-suspended in 1 mL of washing buffer, and then centrifuged at 1,500 g for 5 min at 4 °C. The supernatant was discarded and the precipitate was washed again. Finally, samples were re-suspended in 1 mL hypotonic buffer solution (10 mM HEPES/KOH pH 7.9, 4 mM MgCl_2_, protease inhibitor cocktail -P9599, Sigma, Saint Louis, MO, USA-), centrifuged at 1,500 g for 5 min at 4 °C and re-suspended again in the same buffer. Extracts were shaken for 2 h at 4 °C. Then, chloroplast protein samples were freeze-dried and stored at −80 °C until the proteomic analysis was performed.

### LC-MS/MS differential proteomic analysis

Freeze-dried samples were re-suspended in solubilization buffer (50 mM HEPES pH 7.9, 4% CHAPS, protease inhibitor cocktail P9599) and incubated for 1 h at 4 °C, under agitation and centrifuged at 12,000 g for 15 min. This step was repeated twice, and supernatants separated in each sample were used for a label-free differential proteomic analysis. Three independent biological replicates were analyzed per treatment and ecotype.

Samples (40 µg) were loaded into a 12% SDS-PAGE to prepare them for trypsin digestion (Promega), as described^60^. Then, peptides were loaded onto an analytical column (LC, 3 µm C18-CL, 75 µm × 12 cm, Nikkyo) and analyzed in a mass spectrometer nanoESI qQTOF (5600 TripleTOF, ABSCIEX). Identification of the obtained peaks was carried out with the ProteinPilot software (version 4.5.1, revision 2768; Paragon™ Algorithm 4.5.1.0, 2765; Sciex), using the defaults parameters (trypsin specificity, cys-alkylation, no taxonomy restriction and the search rapid) and the ExPASy database^61^. Protein identification was performed at 95% confidence.

Peptide and protein areas were calculated using Peak View 1.1 (http://sciex.com/products/software/peakview-software) and the quantitative data obtained analyzed with Marker View 1.3 (http://sciex.com/products/software/markerview-software). Statistical analysis (two factor ANOVA and hierarchical clustering) was performed using MeV (MultiExperiment Viewer) from Tm4 platform^62^. The experiment was normalized by amount of protein in each sample, and the differentially abundant proteins between ecotypes, stress treatments or the interaction between both factors were identified (p-value < 0.05). The functional analysis was done using the *Kyoto Encyclopedia of Genes and Genomes* (KEGG)^63^, UniProt^64^, LegumeIP^65^ and Miyakogusa.jp 3.0 (http://www.kazusa.or.jp/lotus/) Functional protein association networks were evaluated with the STRING software (version 10.0)^22^. Differentially abundant proteins between ecotypes, treatments or with interaction between both factors were analyzed separately. The *Arabidopsis thaliana* database was used in the analysis, with a required confidence (score) of 0.4. The confidence score is the approximate probability that a predicted link exists between two proteins in the same metabolic map in the KEGG database. The edges in the corresponding graphs showed the predicted functional links between the different proteins, consisting of eight colour lines each one representing one type of evidence. Avalaible databases used for the prediction of functional networks by the STRING software were: gene co-occurrence (blue lines), co-expression (black lines), curated databases (light blue lines), experimentally determined data (violet lines) and textmining (yellow lines).

### Chloroplast transmission electron microscopy (TEM)

Ultrastructural analysis of chloroplast was carried out in the second developed leaf of 3 plants per treatment and ecotype. One leaf of 1 plant cultivated individually in a pot was considered as an experimental unit. Leaf sections (1 mm^2^) were infiltrated and fixed with 2% glutaraldehyde, in sodium phosphate buffer (pH 7.2–7.4), for 2 h at 4 °C. Then, samples were washed three times with the same buffer to remove the excess of fixative, and secondary fixated with 1% osmium tetroxide for 1 h at 4 °C. Dehydration was performed with alcohols, for their inclusion in an epoxy resin. Ultra-thin sections (90 nm) were contrasted with uranyl acetate and lead citrate, and photographed in a transmission electron microscope JEM 1200 EX II (TEM, JEOL, USA).

Image analysis was performed using the ImageJ software (National Health Institute, MD, USA) in a minimum of 20 photographs per treatment and ecotype. Ultrastructural parameters determined were chloroplasts area (µm^2^) and number and area (µm^2^) of starch granules. For pair comparisons between temperature treatments, in each ecotype, a Student’s t-test (p < 0.05) was performed with Prism 5 (Graph-pad Software, CA, USA).

### Chloroplast ROS detection by confocal microscopy

Confocal microscopy analysis was performed according to Shu *et al*.^66^, with some modifications. Briefly, leaves were cut in 1 mm strips and incubated for 10 min with 25 µM 2′,7′-dichlorofluorescein diacetate (DCF-DA), in 20 mM TrisHCl pH 7.8 buffer solution in darkness. Then, samples were washed twice with 20 mM TrisHCl pH 7.0 buffer solution and exposed to a 250 µmol m^−2^ s^−1^ light intensity for 1 min, before observation in a confocal microscope LEICA TCS SP5 (Leica, Germany). As fluorescence controls, leaves were incubated with buffer solution without DCF-DA, or with DCF-DA but without light exposition. During the observation, samples were excited at 485 nm (λ_exc_ DCF-DA, λ_em_ = 535 nm) and 488 nm (λ_exc_ chlorophyll autofluorescence, λ_em_ = 670–730 nm). Images were processed using the Leica LAS AF LITE software (Leica Microsystems CMS GmbH, Germany). Microscopy was performed on the second developed leaf in 4 plants per treatment and ecotype.

### Determination of chloroplast ROS by flow cytometry

Flow cytometer analysis was performed considering an experimental unit as the cell populations isolated from leaves of one plant cultivated individually in a pot. Cells were obtained by partial homogenization of *L*. *japonicus* MG-1 and MG-20 leaves. Two hundred mg of leaves were homogenized in 2 mL homogenization buffer solution (50 mM HEPES pH 7.0, 330 mM sorbitol, 2 mM EDTA, 1 mM MgCl_2_, 5 mM ascorbic acid, 0.05% m/v BSA and 0.5 mM PMSF). The homogenate was filtered with gauze and centrifuged at 1,500 g for 7 min at 4 °C. The supernatant was discarded and the pellet re-suspended in 1 mL of homogenization buffer and diluted 1:8 with the sheath fluid FACSFlowTM (BD Biosciences, CA, USA) for the analysis. Determinations were done using a FACSCalibur flow cytometer (BD Biosciences, Singapore) at the same voltage and at a constant flow rate (<500 events s^−1^) for all experiments. BD CellQuest software (BD Biosciences, CA, USA) was used for data acquisition. Cytograms analysis was performed using the FlowJo software (TreeStar Inc., CA, USA).

The flow cytometer was equipped with a red Diode laser (λ_em_ = 635), which allows acquisition of 6 different parameters simultaneously: 4 colours (FL1, FL2, FL3 and FL4) and two parameters related with light scattering, side scatter (SSC) and the forward scatter. In this study only the FL1, FL3 and SSC parameters were used. FL1 (λ_exc_ = 488 nm, λ_em_ = 530 ± 30 nm BP) allows the detection of DCF-DA, that emits in green when excited with blue light. The FL3 parameter allows the detection of chlorophyll autofluorescence after excitation with blue light (λ_exc_ = 488 nm, λ_em_ = 670 nm LP). SSC represents the scattered light by particles at an angle of 90°, and it is related with the internal complexity of the cell and their size.

Cytometric populations obtained from *L*. *japonicus* leaves were characterized, physically separated by the flow cytometer and identified. Briefly, populations were filtered through a 0.22 µm polycarbonates filters (Millipore, MA, USA), DAPI stained and observed in an epifluorescence microscope Nikon Eclipse E600 (Nikon Instruments, NY, USA).

Treatments were done incubating samples with 10 µM DCF-DA in the darkness for 10 min, before exposure to 250 µmol m^−2^ s^−1^ of light for 1 min. Then, the flow cytometer fluorescence determination was performed. As fluorescence controls, samples were incubated with buffer solution without DCF-DA, or with DCF-DA but without light exposition. To reduce variability between incubation treatments, these were analyzed sequentially in each tube (first without DCF-DA, then with DCF-DA in darkness and finally with DCF-DA and light exposition).

In each case, the FL1 mode was determined for the cytometric population selected (40,000 events), and the difference between the DCF-DA_light_ and DCF-DA_darkness_ treatments calculated for this parameter. As the FL1 fluorescence was registered in a logarithmic scale, the difference between the modes in each treatment was calculated as follow:

1 $${{\rm{FL1}}}_{{\rm{Sample}}}=\,\mathrm{Log}({{\rm{FL1}}}_{{\rm{DCF}}-{\rm{DA}}\_{\rm{light}}})-\,\mathrm{Log}({{\rm{FL1}}}_{{\rm{DCF}}-{\rm{DA}}\_{\rm{darkness}}})$$

The obtained results were compared between control and stress treatments for each ecotype. Measurements were performed in 5 plants per treatment and ecotype. The FL1 values are expressed as arbitrary units (UAs). For the statistical analysis, one-way ANOVA was carried out, followed by a Tukey test for multivariate analysis (p < 0.05) using Prism 5 (Graph-pad Software, CA, USA) and InfoStat/L program (Universidad Nacional de Córdoba, Córdoba, Argentina)^67^. For pair comparisons between temperature treatments, a Student’s t-test (p < 0.05) with Prism 5 (Graph-pad Software, CA, USA) was performed.

### One sentence summary

Acclimation responses in two *Lotus japonicus* ecotypes with different tolerance to cold stress are related to global chloroplast changes that involve its proteome, ultrastructure and ROS levels.

## Supplementary information

Supplementary material
