# Supplementary material for: The increase of photosynthetic carbon assimilation as a mechanism of adaptation to low temperature in Lotus japonicus
Source: Sci Rep. 2019 Jan 29;9:863. doi: 10.1038/s41598-018-37165-7 (PMC6351645; doi:10.1038/s41598-018-37165-7)
Supplement: Supplementary file 1 — Supplementary material [file 41598_2018_37165_MOESM1_ESM.pdf]

The increase of photosynthetic carbon assimilation as a mechanism of adaptation to low temperature in *Lotus japonicus*

Calzadilla, Pablo Ignacio; Vilas, Juan Manuel; Escaray, Francisco José; Unrein, Fernando; Carrasco, Pedro; Ruiz, Oscar Adolfo.

Supplementary Table 1: Functional assignation of the differentially abundant proteins with significative interaction between ecotypes and temperature treatments. The classification and functional assignation of the significant abundant proteins ($p<0.05$) was done using KEGG, LegumeIP and Uniprot databases. Values are the mean ± SD of three biological replicates, and are expressed as arbitrary units (AUs) relative to the MG-1 control treatment. 1C, MG-1 Control; 1L, MG-1 Low temperature; 20C, MG-20 Control; 20L, MG-20 Low temperature.

Id Lj	Uniprot	# KO	KEGG Annotation	Expression pattern
Lj3g3v3032110.2	>sp|P11894|RK9_PEA	K02939	large subunit ribosomal protein L9	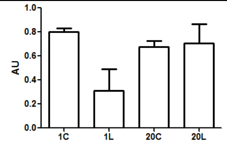
Lj6g3v0925890.1	>sp|B6U151|GATA_MAIZE	K02433	aspartyl-tRNA(Asn)/glutamyl-tRNA(Gln) amidotransferase subunit A [EC:6.3.5.6]	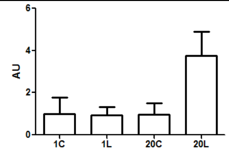
Lj1g3v4154900.1	>sp|Q69RJ0|GLTB_ORYSJ	K00284	glutamate synthase (ferredoxin) [EC:1.4.7.1]	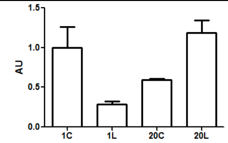
Lj2g3v1002750.2	>sp|P17067|CAHC_PEA	K01673	carbonic anhydrase [EC:4.2.1.1]	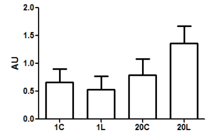
Lj0g3v0100069.1	>sp|P46285|S17P_WHEAT	K01100	sedoheptulose-bisphosphatase [EC:3.1.3.37]	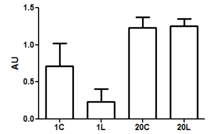
Lj5g3v2045360.1	>sp|P93735|ELIP1_ARATH	-----	Chlorophyll a-b binding	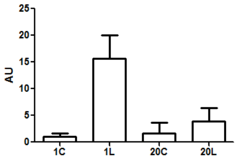
Lj0g3v0050739.1	>sp|Q9BBU3|PSBA_LOTJA	K02703	photosystem II P680 reaction center D1 protein [EC:1.10.3.9]	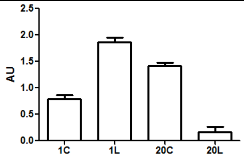
Lj0g3v0160239.1	>sp|Q9BBQ5|PETD_LOTJA	K02637	cytochrome b6-f complex subunit 4	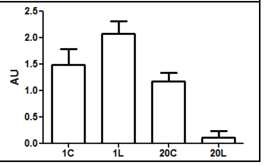

Lj0g3v0341139.1	sp|P31853|ATPX_SPIOL	K02109	F-type H+-transporting ATPase subunit b [EC:3.6.3.14]	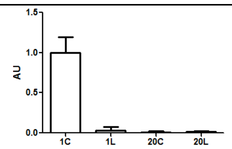
Lj2g3v0609720.1	>sp|Q6DQL1|SUCA2_SOL LC	K01899	succinyl-CoA synthetase alpha subunit [EC:6.2.1.4 6.2.1.5]	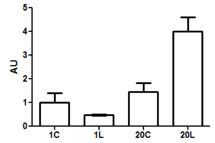
Lj2g3v0609720.1	>sp|Q6ZL94|SUCA_ORYS J	K01899	succinyl-CoA synthetase alpha subunit [EC:6.2.1.4 6.2.1.5]	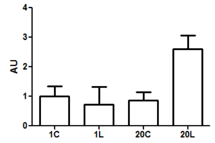
Lj6g3v2133230.1	>sp|Q38732|DAG_ANTM A	K06236	collagen, type I/II/III/V/XI/XXIV/XXVII, alpha	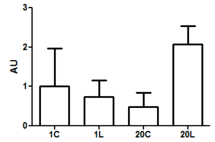

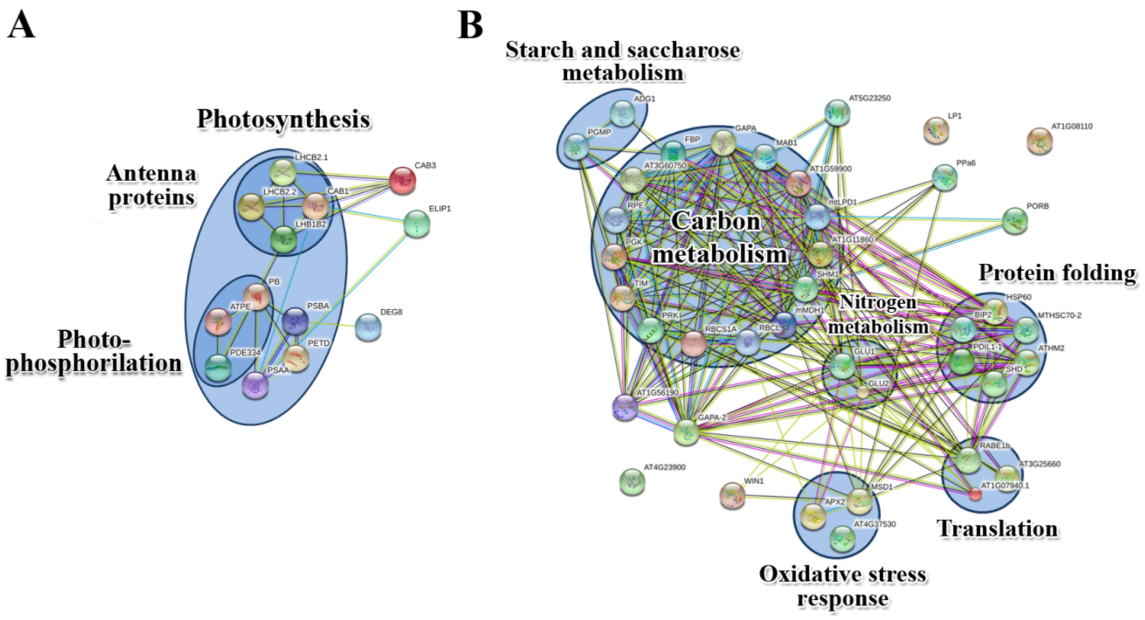

Supplementary Figure 1: Functional association networks of differentially abundant proteins between MG-1 and MG-20 chloroplasts. **A.** Higher abundant proteins in MG-1 compared to MG-20. **B.** Higher abundant proteins in MG-20 compared to MG-1. Data from gene co-occurrence (blue lines), co-expression (black lines), curated databases (light blue lines), experimentally determined (violet lines) and textmining (yellow lines) were used as the prediction methods in the STRING software. The confidence value used for the analysis was *Score* = 0.4.

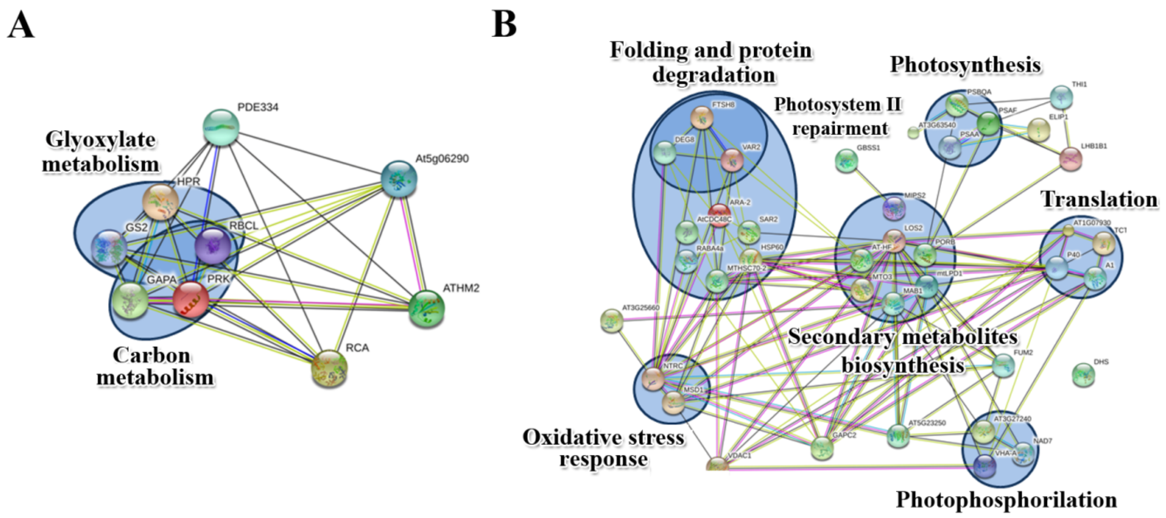

Supplementary Figure 2: Functional association networks of differentially abundant proteins between temperature treatments. **A.** Higher abundant proteins in control compared to low temperature treatment. **B.** Higher abundant proteins in the low temperature treatment compared to control. Data from gene co-occurrence (blue lines), co-expression (black lines), curated databases (light blue lines), experimentally determined (violet lines) and textmining (yellow lines) were used as the prediction methods in the STRING software. The confidence value used for the analysis was *Score* = 0.4.

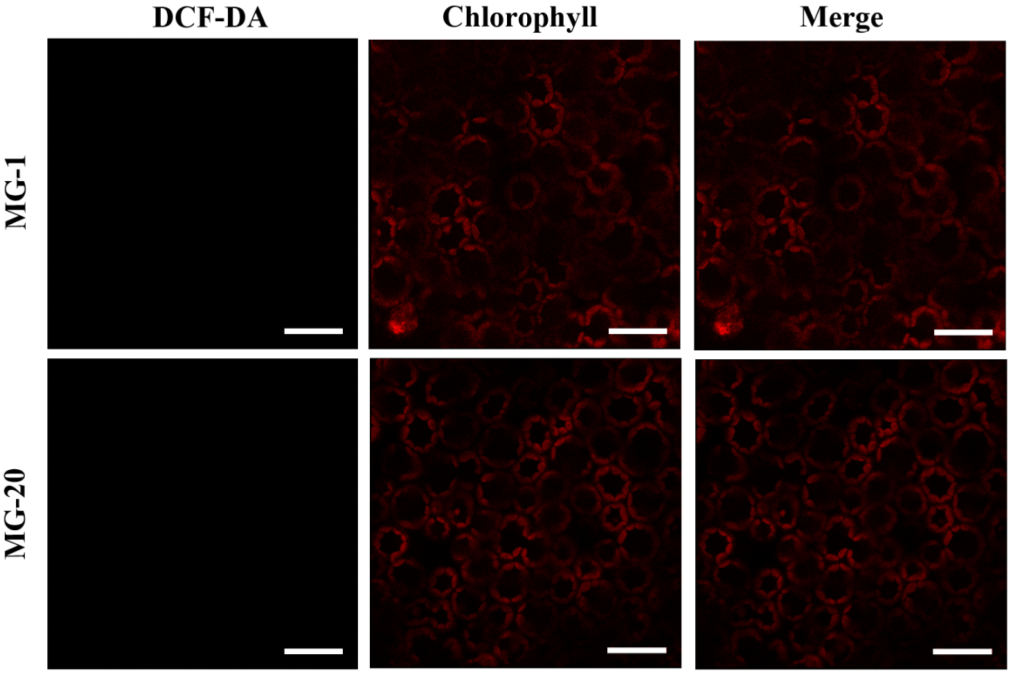

Supplementary Figure 3: Absence of green fluorescence in leaves of L. japonicus not incubated with DFC-DA. Representative images of laser confocal microscopy. Scale bars = 30 μm.

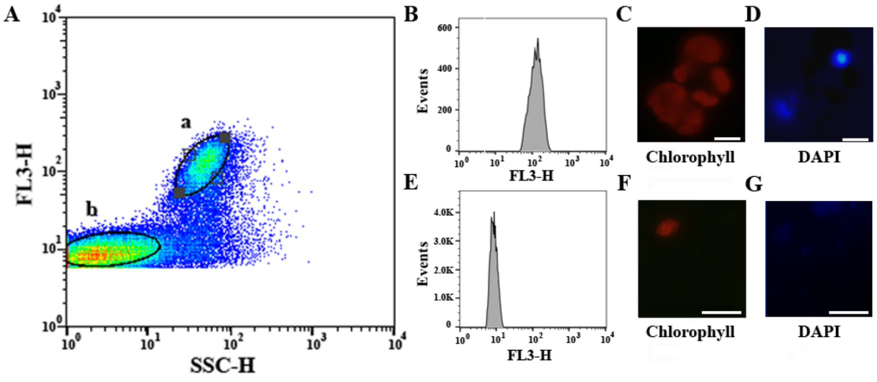

Supplementary Figure 4: Characterization of cells population extracted from L. japonicus leaves. **A.** SSC vs FL3 cytogram of isolated cells obtained from *L. japonicus* leaves. *a*. Intact cells population. *b*. isolated chloroplasts population and cellular debris. **B.** and **E.** Representative histograms of FL3 fluorescence intensity of *a* and *b* population, respectively. **C-D** and **F-G.** Epifluorescence microscope images of *a* and *b* populations, respectively. **C**, **F**; Images showing chlorophyll autofluorescence. **D**, **G**; Images showing the presence of nucleus, after DAPI incubation. Scale bars = 10 µm.

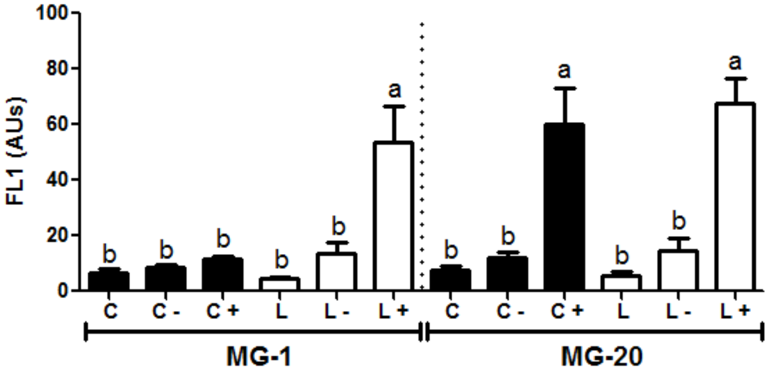

Supplementary Figure 5: ROS production quantification by flow cytometry in L. japonicus isolated cells. Values are the mean ± SD of 5 biological replicates and are expressed as arbitrary units (AUs), for each ecotype and treatment. C, Control; C-, Control cells incubated with DCF-DA but without light; C+, Control cells incubated with DCF-DA and light; L, Low temperature; L-, Low temperature cells incubated with DCF-DA but without light; L+, Low temperature cells incubated with DCF-DA and light. Different letters indicate significant differences between treatments (Tukey test, $p<0.05$).
